# Anti-HLA Class II Antibodies Are the Most Resistant to Desensitization in Crossmatch-positive Living-donor Kidney Transplantations: A Patient Series

**DOI:** 10.1097/TXD.0000000000001695

**Published:** 2024-08-29

**Authors:** Annelies E. de Weerd, Dave L. Roelen, Michiel G.H. Betjes, Marian C. Clahsen-van Groningen, Geert W. Haasnoot, Marcia M.L. Kho, Marlies E.J. Reinders, Joke I. Roodnat, David Severs, Gonca E. Karahan, Jacqueline van de Wetering

**Affiliations:** 1 Department of Internal Medicine, Erasmus MC Transplant Institute, University Medical Center, Rotterdam, The Netherlands.; 2 Department of Immunology, Leiden University Medical Center, Leiden, The Netherlands.; 3 Department of Pathology, Erasmus MC Transplant Institute, University Medical Center, Rotterdam, The Netherlands.

## Abstract

**Background.:**

In HLA-incompatible kidney transplantation, the efficacy of desensitization in terms of anti-HLA antibody kinetics is not well characterized. We present an overview of the course of anti-HLA antibodies throughout plasma exchange (PE) desensitization in a series of crossmatch-positive patients.

**Methods.:**

All consecutive candidates in the Dutch HLA-incompatible kidney transplantation program between November 2012 and January 2022 were included. The eligibility criteria were a positive crossmatch with a living kidney donor and no options for compatible transplantation. Desensitization consisted of 5–10 PE with low-dose IVIg.

**Results.:**

A total of 16 patient-donor pairs were included. Patients had median virtual panel-reactive antibody of 99.58%. Cumulative donor-specific anti-HLA antibody (cumDSA) mean fluorescence intensity (MFI) was 31 399 median, and immunodominant DSA (iDSA) MFI was 18 677 for class I and 21 893 for class II. Median anti-HLA antibody MFI response to desensitization was worse in class II as compared with class I (*P* < 0.001), particularly for HLA-DQ. Class I cumDSA MFI decreased 68% after 4 PE versus 53% in class II. The decrease between the fifth and the 10th PE sessions was modest with 21% in class I versus 9% in class II. Antibody-mediated rejection occurred in 85% of patients, with the iDSA directed to the same mismatched HLA as before desensitization, except for 3 patients, of whom 2 had vigorous rebound of antibodies to repeated mismatches (RMMs). Rebound was highest (86%) in RMM-DSA with prior grafts removed (transplantectomy n = 7), lower (39%) in non-RMM-DSA (n = 30), and lowest (11%) for RMM-DSA with in situ grafts (n = 5; *P* = 0.018 for RMM-DSA transplantectomy versus RMM-DSA graft in situ). With a median follow-up of 59 mo, 1 patient had died resulting in a death-censored graft survival of 73%.

**Conclusions.:**

Patients with class II DSA, and particularly those directed against HLA-DQ locus, were difficult to desensitize.

## Graphical abstract

**Figure:**
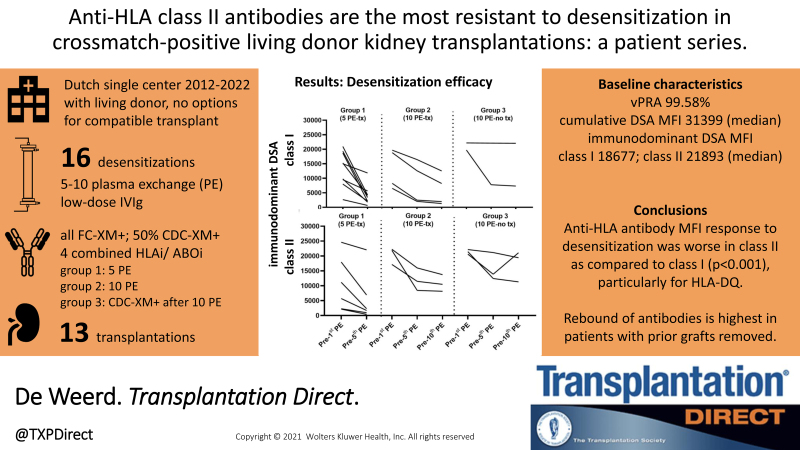

Kidney transplantation is the treatment of choice for patients with end-stage kidney disease. Circulating donor-specific anti-HLA antibodies (DSAs) are a major hurdle in kidney transplantation because they can lead to antibody-mediated rejection (AMR) and accelerated graft loss.^1^ Sensitized patients with anti-HLA antibodies against the vast majority of potential donors have lower access to transplantation with increased morbidity and mortality on the waitlist.^2,3^ Kidney exchange and priority programs for highly sensitized patients increase the chance of an HLA-compatible donor offer, but especially patients with virtual panel-reactive antibodies (vPRA) ≥98% accumulate on the waitlist.^4-7^ Desensitization, that is, preoperative removal of HLA antibodies, is therefore a viable option for patients with very low chances of an HLA-compatible donor offer. Although AMR remains to occur frequently and outcomes are inferior as compared with HLA-compatible transplantation, desensitization may be the only option for transplantation in this patient group.^8-10^

Although desensitization can be performed by different methods, the backbone of most therapies is a form of apheresis combined with IVIg with or without rituximab.^11,12^ Other approaches are complement inhibition, antiplasma cell and interleukin-6 therapy.^13-16^ There is only a limited number of randomized controlled trials performed in this patient group,^13,17,18^ and currently, a randomized controlled trial with the novel drug imlifidase is recruiting patients in the United States. Although these studies report the clinical outcomes after HLA-incompatible (HLAi) kidney transplantation, data on the efficacy of desensitization procedures are scarce.^19,20^ Noble et al^19^ compared 3 apheresis techniques in 45 patients who were desensitized by either immunoadsorption (IA), plasma exchange (PE), or double-filtration plasmapheresis (DFPP). The mean overall reduction in mean fluorescence intensity (MFI) of class I DSA was 88% and of class II was 59%, with higher efficacy with IA and PE than with DFPP.^19,20^

We have performed living-donor HLAi kidney transplantation in our national referral center in patients whose options for compatible transplantation were exhausted. Of note, when discussing HLAi transplantation, a recent systematic review identified a high level of heterogeneity in defining HLA incompatibility: complement-dependent cytotoxicity (CDC) versus flow cytometry (FC) versus virtual crossmatches (XMs).^21^ In our desensitization program, the sensitization status was reflected in vPRA approaching 100% and an initial positive FC-XM in all and CDC-XM in half of patients. Our protocol with PE required a negative CDC-XM to proceed with transplantation irrespective of the result of the Luminex bead-based antibody detection assay.

The aim of the current study was to investigate the efficacy of desensitization by PE in terms of decrease in MFI of anti-HLA antibodies, including the cumulative DSA (cumDSA) and immunodominant DSA (iDSA), as well as the predominant class of DSA and the rebound in the first postoperative month.

## MATERIALS AND METHODS

### Study Cohort

Desensitization for living-donor HLAi kidney transplantation is centralized in one national referral center (Erasmus MC), and is eligible only for patients whose options for HLA-compatible kidney transplantation with either a deceased or living donor were exhausted. All consecutive adult patients with end-stage kidney disease who underwent desensitization between November 2012 and January 2022 were included. Eligibility criteria for the living-donor HLAi kidney transplantation program were vPRA ≥85%, low chances of finding a compatible donor defined as either ≥2 y of participation in the Eurotransplant Acceptable Mismatch program or ≥1 y of participation in the national kidney exchange program, and a positive T-cell FC-XM or CDC-XM with a living donor. Combined ABO blood group and HLA incompatibility was allowed. Written informed consent was obtained from all patients. This trial was approved by the institutional review board of the Erasmus Medical Center (MEC-2021-0357). The clinical and research activities being reported are consistent with the Principles of the Declaration of Istanbul as outlined in the “Declaration of Istanbul on Organ Trafficking and Transplant Tourism” and the Declaration of Helsinki as outlined in the “Ethical Principles for Medical Research Involving Human Subjects.”

### Desensitization and Immunosuppression Protocol

The desensitization protocol consisted of 5 or 10 PE sessions (1.5 times plasma volume exchange). In a period of 7 d, 5 PE sessions were performed at days 1, 3, 5, 6, and 7. IVIg was administered 0.1 g/kg after each PE. One week before surgery, PE was commenced together with tacrolimus 0.2 mg/kg/d, mycophenolate mofetil 2000 mg/d, and prednisolone 20 mg/d. After surgery, PE was performed at posttransplant days 2, 4, and 6. Induction therapy consisted of rabbit antithymocyte globulin: 1.0 mg/kg given 1 d before transplantation and 1.5 mg/kg daily at days 1–4 after transplantation. In the case of ABO-incompatibility, alemtuzumab 30 mg was administered subcutaneously instead of rabbit antithymocyte globulin. Target trough blood concentrations for tacrolimus were 8–12 µg/L in the first 3 mo and 5–8 µg/L thereafter; target mycophenolate mofetil concentrations were 1.5–3.0 mg/L. Prednisolone was lowered to 10 mg by 1 mo and tapered to 5.0–7.5 mg/d from 3 mo onward. BK viremia was routinely monitored for by month 3, and if present, mycophenolate mofetil dosing was adjusted accordingly.

For-cause biopsies were performed in the case of deteriorating kidney function or proteinuria. Histology was assessed according to revised 2017 Banff criteria.^22^ AMR was treated with pulse methylprednisolone 1000 mg daily for 3 consecutive days and 5 PE combined with IVIg 2 g/kg. If refractory, alemtuzumab 30 mg subcutaneously was added. Chronic-active AMR (ca-AMR) was treated with pulse methylprednisolone 1000 mg daily for 3 consecutive days combined with IVIg 1 g/kg. As of late 2017, in the case of refractory ca-AMR, tocilizumab 8 mg/kg monthly was initiated.^23^

### Anti-HLA Antibody Analysis and DSA Assignment

Pretransplant serum samples were collected before the first PE, the fifth PE and, if applicable, the 10th PE. These samples were drawn before induction therapy. For the current analysis, posttransplant serum samples were defined as those collected within 1 mo after transplantation.

All serum samples were pretreated with ethylenediaminetetraacetic acid, at a final concentration of 8 mM and tested using Luminex HLA class I and II SAB kits (LabScreen; One Lambda, Inc, Canoga Park, CA).^24^ Briefly, 20 μL serum was mixed with 4 μL class I or II beads and further tested as recommended by the manufacturer. Data were analyzed using HLA FUSION antibody software version 3.4.18 (One Lambda, Inc). Results were expressed as baseline (normalized) MFI, and 1000 MFI was applied as cutoff. DSA assignment for each patient was performed at the split antigen level for HLA-A, -B, -DR, and -DQ, upon consideration of antibody reactivity patterns. In the case of multiple beads corresponding to the same split antigen specificity, results were expressed as average MFI of multiple bead MFIs.

### XM Tests

XMs were performed using (unseparated) total peripheral blood mononuclear cells, by both CDC and FC methods before the first, fifth, and the 10th PE. Decision to proceed with transplantation was based on conversion of positive CDC-XM to negative.

CDC-XM was performed in the presence and absence of dithiothreitol using both undiluted and 1:4 diluted sera, as described earlier.^25^ Cell lysis >27% in dithiothreitol added wells was considered a positive IgG CDC-XM.

FC-XM was performed using standard FC-XM protocols, as previously described.^26^ T-cell FC-XM was deemed positive with an MFI ratio of >1.6, whereas B-cell FC-XM was considered positive with an MFI ratio of >2.6.

### Statistical Analysis

Patients were divided into three groups: 5 PE (group 1) and 10 PE (group 2) sessions who proceeded with transplantation, and patients who experienced no seroconversion of the CDC-XM despite 10 PE sessions, and hence no transplantation (group 3). Categorical data are presented as numbers and percentages. Continuous data are shown as median and ranges. Mann-Whitney U test was used to compare differences between groups. Graft survival was analyzed using the Kaplan-Meier survival method. DSA with the highest MFI was termed iDSA, and the sum of all DSA MFI was termed cumDSA. Graphpad Prism (version 9.0.1) was used to visualize the data.

## RESULTS

### Baseline Characteristics of the Study Cohort

A total of 16 patient-donor pairs were included in this study (Table 1). Patients had a median age of 38 y (range 28–69) and 56% were women. Twelve patients (75%) were preparing for a repeat transplantation with 2 (0–6) previous transplantations. The remaining 4 patients without previous transplantation were all women with previous pregnancies. All patients except 1 had a vPRA >99.16% (median: 99.58, range: 93.16–100). Patients were on dialysis for 4.8 y median, except for 2 pre-emptive patients with a failing previous transplantation. Four patients were both HLAi and ABO blood group–incompatible, and all 4 had an incompatible blood group A donor to whom anti-A titers ranged between 1:2 and 1:64 (automated column technique) before desensitization (**Table S1, SDC**, http://links.lww.com/TXD/A689).

**TABLE 1.** Baseline characteristics of the study cohort

	Group 1 (5 PE-tx), n = 9	Group 2 (10 PE-tx), n = 4	Group 3 (10 PE-no tx), n = 3
Patient age, median y (range)	35 (28–69)	43 (33–53)	46 (37–68)
Sex, female	4	3	2
Underlying renal disease			
Diabetic nephropathy	1	0	0
IgA nephropaty	0	0	1
Systemic lupus erythamotosus nephritis	1	0	0
Atypical hemolytic uremic syndrome	1	0	0
Nephronophthisis	1	0	0
Focal segmental glomerular sclerosis	2	0	1
Obstructive nephropathy	2	3	1
Etiology unknown	1	1	0
Dialysis modality			
Hemodialysis	6	4	3
Peritoneal dialysis	1	0	0
Pre-emptive with failing previous donor kidney	2	0	0
Dialysis vintage, mo, median (range)	44 (0–120)	59 (11–82)	173 (14–177)
Previous transplantations	2 (0–6)	1 (0–2)	1 (0–2)
Known sensitizing events			
Previous transplantations, number of patients (%)	8 (89)	2 (50)	2 (67)
Pregnancies, number of patients (%)	3 (33)	2 (50)	1 (67)
vPRA (%, average)	99.18	99.77	99.86
Donor characteristics			
Age, y, median (range)	52 (31–61)	56 (45–60)	NA
Living-related	6	3	NA
Living-unrelated	3	1	NA
HLA-incompatible	7	2	NA
HLA- and ABO blood group–incompatible	2	2	NA
Number of HLA mismatches (A, B, C, DR1/3-5, DQ), median (range)			
All	7 (4–9)	5 (3–6)	10 (9–10)
Class I	3 (2–6)	3 (1–3)	5 (5–6)
Class II	3 (1–4)	2 (2–3)	4 (4–5)
DSA status (n, patients)			
Only class I	3	1	0
Only class II	0	0	1
Classes I and II	6	3	2
Immunodominant DSA			
Class I	7	1	1
Class II	2	3	2

DSA, donor-specific antibody; NA, not available; PE, plasma exchange; tx, transplantation; vPRA,virtual panel-reactive antibody.

Patients had a median of 5 HLA class I (HLA-A, -B, and -C) and 5 class II (HLA-DR and -DQ) mismatches. Before desensitization, a total of 57 DSA with a median MFI of 10 693 (range 1180–24 612) were detected, 33 of which were directed at HLA class I and 24 were directed at HLA class II (Table 2). The majority of patients had combined class I and II DSA (69%), 25% had only class I, and 6% had only class II DSA. The iDSA was of class I in 56% of patients, however, with lower median MFI for class I (18 677) than for class II (21 893) (Table 2).

**TABLE 2.** Donor-specific antibody status before desensitization

Patient no.	Study group	DSA class I (MFI)	DSA class II (MFI)	CDC-XM	T-cell FC-XM (ratio)	B-cell FC-XM (ratio)
p1	Group 1	A33 (3723), B44 (8007)	−	−	+ (4.18)	n.i
p3	Group 1	A3 (2657)	DQ6 (2174)	−	+ (1.89)	n.i
p4	Group 1	B18 (10 693), B62 (19 330)	−	−	+ (5.26)	+ (3.11)
p6	Group 1	A3 (9543), B7 (5540), B44 (3826)	DR51 (2282)	−	+ (2.9)	+ (2.65)
p8	Group 1	A1 (13 704), Cw6 (15 005)	DR1 (2396), DQ5 (24 612), DQ9 (16 177)	−	+ (4.15)	+ (7.54)
p9^*a*^	Group 1	A3 (6048), A24 (9784), B7 (8408), B35 (9172)	DR13 (5720), DR52 (4342), DQ6 (2301)	−	+ (7.86)	+ (15.1)
p10	Group 1	A68 (15 259), B44 (12 539), Cw4 (3863)	DR13 (2914), DR53 (17 952), DQ6 (3610)	+	+ (6.95)	+ (10.53)
p11^*a*^	Group 1	B8 (18 677)	−	−	+ (8.05)	+ (7.94)
p13	Group 1	B42 (20 974), Cw17 (5756)	DR11 (11 140), DR52 (11 172)	−	+ (2.78)	+ (2.88)
p2	Group 2	A25 (12 484), B44 (18 847)	−	+	+ (27.29)	+ (5.25)
p5B^*a*^	Group 2	A24 (8266), Cw7, (1308)	DQ5 (21 893)	+	+ (7.5)	+ (5.35)
p7^*a*^	Group 2	B60 (6529)	DR12 (17 173)	+	+ (2.89)	+ (15.96)
p12	Group 2	B58 (19 687)	DR13 (20 474), DQ7 (22 270)	+	+ (17.8)	+ (15.5)
p5A	Group 3	A1 (22 196), A2 (20 429), B49 (1180), Cw7 (2355)	DR8 (6324), DR13 (11 617), DQ4 (13 391), DQ6 (20 607)	+	+ (17.82)	+ (5.86)
p14	Group 3	−	DQ6 (22 159)	+	(1.39)	+ (12.1)
p15	Group 3	B7 (19742), B8 (17 362), Cw7 (4037)	DR17(10245), DQ6 (21 464)	+	+ (13.69)	+ (25.68)

^*a*^Combined HLA- and ABO blood group–incompatible transplantation.

CDC-XM, complement-dependent cytotoxicity crossmatch; FC-XM, flow cytometric crossmatch; MFI, mean fluorescence intensity; n.i, not interpretable because of low B-cell count.

Before the start of desensitization, CDC-XM in current serum was positive in 8 (50%), whereas FC-XM was positive in all patient-donor pairs (Table 2). The median cumDSA were 31 399 and the iDSA 19 089, respectively.

MFI values of HLA mismatches with current donor candidates, prior allografts (transplantectomy versus graft in situ) as well as children are detailed in Table S2 (**SDC**, http://links.lww.com/TXD/A689). The vast majority of patients (88%) had repeated mismatches (RMMs) that could be linked to immunizing events. Only in 4 of these 14 patients (29%) with documented RMMs, this RMM was the iDSA.

### Effect of Desensitization on Anti-HLA Antibodies

**FIGURE 1.**
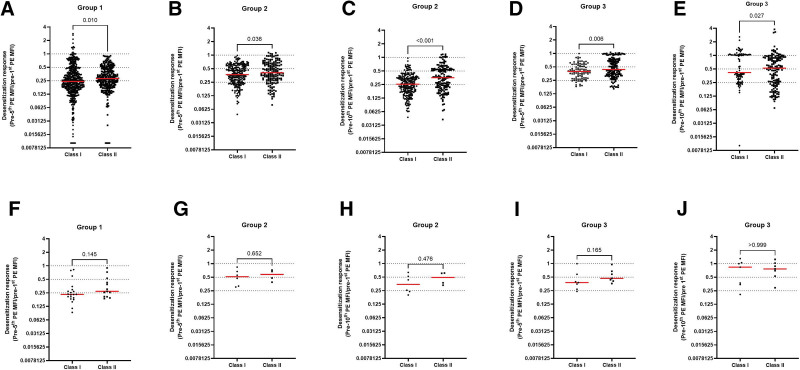
Class I and class II MFI response to desensitization. The response to desensitization as detected by Luminex single-antigen bead assay is depicted for the total of anti-HLA antibodies (A)–(E) as well for DSA (F)–(J). Patients were divided into 3 groups according to desensitization outcome: 5 PE and transplanted (group 1, n = 9); 10 PE and transplanted (group 2, n = 4); and 10 PE and not transplanted (group 3, n = 3). Predesensitization, only 1 of 9 patients had CDC-XM positivity in group 1, whereas all patients in groups 2 and 3 were CDC-XM positive. After desensitization, all patients in groups 1 and 2 had negative CDC-XM and hence were transplanted, whereas in group 3 CDC-XM remained positive precluding transplantation. Red lines indicate median values. Mann-Whitney U test was used to compare differences between groups. A *P* value of <0.05 was considered significant. In the analysis of groups 1, 2 and 3 combined, median anti-HLA antibody MFI response to desensitization was worse in class II as compared to class I (*P* < 0.001). Analyses on DSA followed the same trend; however, no statistical significance was reached (*P* = 0.05). CDC-XM, complement-dependent cytotoxicity crossmatch; DSA, donor-specific antibody; MFI, mean fluorescence intensity.

To assess the effect of desensitization on Luminex single-antigen bead MFI values, we calculated a response-to-desensitization ratio by dividing postdesensitization MFI with predesensitization MFI value. Median anti-HLA antibody MFI response to desensitization was worse in class II in comparison with class I in all 3 groups (*P* < 0.001, Figure 1A–E). In addition, although group 1 and group 2 were comparable (*P* > 0.05) in response to desensitization for class I, group 3 responses were significantly worse than group 1 (*P* < 0.001) and group 2 (*P* < 0.001). The class II MFI response to desensitization was better in group 1 than in group 2 and group 3 (*P* < 0.05, *P* < 0.007, *P* < 0.05). Analyses on DSA followed the same trend; however, no statistical significance was reached, possibly because of a low number of data points (*P* = 0.05, Figure 1F–J). Noteworthy, neither group 2 nor group 3 had improved responses for class II after the first 4 PE sessions, whereas group 2 benefitted from longer PE sessions for class I. This improvement in class I with longer desensitization aligns with conversion of positive CDC-XM to negative at the end of ninth PE in group 2.

### Effect of Desensitization on DSA and XM Results

**FIGURE 2.**
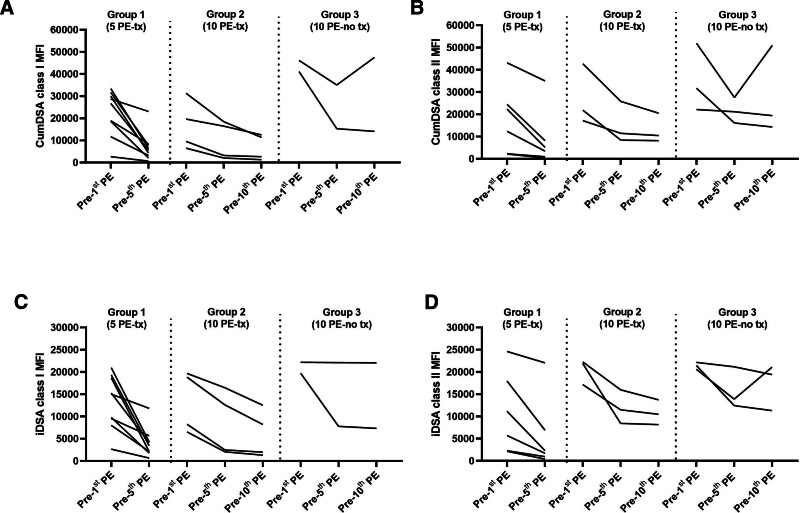
Changes in cumDSA and iDSA MFI values for classes I and II before and after desensitization. Patients were divided into three groups according to desensitization outcomes: 5 PE and transplanted (group 1, n = 9); 10 PE and transplanted (group 2, n = 4); and 10 PE and not transplanted (group 3, n = 3). Predesensitization, only 1 of 9 patients had CDC-XM positivity in group 1, whereas all patients in groups 2 and 3 were CDC-XM positive. After desensitization, all patients in groups 1 and 2 had negative CDC-XM and hence were transplanted, whereas in group 3 CDC-XM remained positive precluding transplantation. The decrease in MFI of cumDSA was higher for class I (A) than for class II (B), in line with the higher decrease of the immunodominant DSA in class I (C) than for class II (D). cumDSA, cumulative DSA; DSA, donor-specific antibody; iDSA, immunodominant DSA; MFI, mean fluorescence intensity; PE, plasma exchange; tx, transplantation.

After desensitization, 5 of 8 patients with positive CDC-XM converted to negative and proceeded to transplantation, whereas 3 remained positive and were not transplanted (group 3). CumDSA MFI values decreased from a median of 31 399 before desensitization to 11 118 after desensitization, reflecting T-cell ratios decreasing from 13.69 to 2.62 and B-cell FC-XM ratios from 25.68 to 7.94 (Table 2 and **Figure S1, SDC,** http://links.lww.com/TXD/A689). Class I cumDSA decreased by 68% before the fifth PE versus 53% for class II, and by 21% versus 9% between the fifth and the 10^th^ PE for classes I and II, respectively (Figure 2A and B). All predesensitization iDSA maintained the highest MFI after desensitization (**Table S2, SDC**, http://links.lww.com/TXD/A689).

Group 1 (n = 9) and group 2 (n = 4) patients had negative CDC-XM before the 5th or 10th PE, respectively, and proceeded with transplantation. One group 3 patient (p5A) was re-enrolled with a different donor (p5B in group 2) after 3 y. The decrease in cumDSA versus iDSA MFI for both classes I and II showed the highest reduction in group 1, lower in group 2, and the lowest din group 3 (Figure 2 and **Figure S2, SDC**, http://links.lww.com/TXD/A689).

### Serial Dilutions of CDC-XM-positive Predesensitization Sera

Although patients in groups 2 and 3 had comparable median iDSA MFI before desensitization, 20 370 and 22 159, respectively, conversion of a positive CDC-XM to negative was achieved only in group 2. Noteworthy, the saturation of beads, particularly in highly sensitized patient samples, may hamper accurate assessment when antibody titers are high titer. To overcome this issue, and with the aim to be able to predict which patients would benefit from desensitization treatments, we compared 4-fold serial dilutions of the predesensitization sera of 3 patients in group 2 with positive CDC-XM with 3 patients who failed desensitization (group 3). Figure S3 (**SDC**, http://links.lww.com/TXD/A689) shows the changes in titer in DSA of the predesensitization samples. Interestingly, in group 2, 4 (57%) of 7 DSA had high initial titers, whereas, in group 3, only 4 (57%) of 14 DSA were high titer antibodies (>1024), and thereby high titers were not discriminative for seroconversion of the CDC-XM.

### Posttransplant DSA and Clinical Outcomes

In total, 13 patients proceeded with transplantation after 5 or 10 PE. A total of 10 de novo or newly detected DSA were detected in 5 (38%) patients within the first month after transplantation (Table 3). In 3 of 5 patients with newly detected DSA (p3, p4, p6, p7, and p13), the antibodies concerned RRMs (p3, p4, and p7). All newly detected DSA had lower MFI than pre-existing DSA, and 9 were lower than MFI 2241.

**TABLE 3.** Newly detected^*a*^ donor-specific HLA antibodies within the first month after HLA-incompatible kidney transplantation

Patient no.	Historic	Predesensitization	Postdesensitization	Newly detected^*a*^ class I DSA (MFI)	Newly detected^*a*^class II DSA (MFI)	Detection within post-tx month 1	Biopsy-proven AMR	Follow-up, mo
p3	B7 (818) DR13 (303)	B7 (460) DR13 (241)	B7 (0) DR13 (168)	**B7**^*a*^ **(10 040**)	**DR13 (1343**)	Day 28	Post-tx day 8	110
p4	DR9 (1290) DQ5 (533)	DR9 (862) DQ5 (386)	DR9 (42) DQ5 (0)	—	**DR9 (1773**) **DQ5 (1413**)	Day 7	Post-tx day 7	82
p6	Cw5 (0) DR15 (90)	Cw5 (27) DR15 (72)	Cw5 (270) DR15 (176)	Cw5 (1825)	DR15 (1200)	Day 8	Post-tx day 8	75
p7	A24 (126) Cw10 (197) DR52 (1143)	A24 (124) Cw10 (108) DR52 (905)	A24 (199) Cw10 (143) DR52 (690)	A24 (1379) **Cw10 (1327**)	**DR52 (2241**)	Day 12	Post-tx day 11	55
p13	A3 (0)	A3 (57)	A3 (12)	A3 (1052)	—	Day 27	Post-tx day 27	16

Antibodies to repeated mismatches are in bold script.

^*a*^These newly detected DSA were detected in the first postoperative month and could indicate a memory alloimmune response, especially in the case of repeated mismatches (bold).

AMR, antibody-mediated rejection; DSA, donor-specific antibody; MFI, mean fluorescence intensity.

The iDSA before desensitization prevailed during AMR in these 5 patients with iDSA of median 16 366 (range 11 268–22 880). The only exception was p3 with a newly detected DSA of 10 040, albeit lower than the (pre-existing) iDSA of 12 615 at the time of ABMR. Patient 3 had 6 prior transplantations, and although his newly detected HLA-B7 was not a RMM at the antigen level, it shared an epitope with mismatches from previous donors (**Table S2, SDC**, http://links.lww.com/TXD/A689).

Biopsy-proven AMR was diagnosed in 11 (85%) patients, at a median of 8 d posttransplant in patients with 5 PE versus 18 d posttransplant in patients with 10 PE (Table 4). In the 11 patients with AMR, the postoperative iDSA was 16 336 median and cumDSA 25 302, whereas in 2 patients without AMR, this was 2188 for both iDSA and cumDSA.

**TABLE 4.** Outcomes after HLA-incompatible kidney transplantation

		Group 1 (5 PE) n = 9	Group 2 (10 PE) n = 4
Biopsy-proven AMR		7 (78%)	4 (100%)
Time to AMR, d, median (range)		8 (7–27)	18 (7–41)
Biopsy-proven ca-AMR		5 (56%)	3 (75%)
Time to ca-AMR, mo, median (range)		11 (5–56)	24 (9–51)
Follow-up time, mo, median (range)		61 (23–117)	58 (55–95)
Death with functioning graft		1 (11%)	0
Functioning graft within follow-up, death-censored		6 (75%)	2 (50%)
	Follow-up, mo	Status	eGFR last follow-up
Group 1			
p1	43	No AMR, deceased	29
p3	117	ca-ABMR, monthly tocilizumab	28
p4	81	Failure because of ca-ABMR	—
p6	82	AMR, no ca-AMR	76
p8	49	Failure because of ca-ABMR	—
p9	62	ca-AMR, monthly tocilizumab	21
p10	61	ca-AMR, monthly tocilizumab	15
p11	59	No AMR	50
p13	23	AMR	25
Group 2			
p2	61	Failure because of ca-AMR	—
p5B	95	ca-AMR	33
p7	55	Failure because of ca-AMR	—
p12	55	Ongoing AMR, monthly tocilizumab	30

AMR, antibody-mediated rejection; ca-AMR, chronic-active antibody-mediated rejection; eGFR, estimated glomerular filtration rate; PE, plasma exchange.

In 11 patients with AMR, iDSA at time of AMR diagnosis were pre-existing and also directed at the same antigen as the iDSA predesensitization, except for 3 patients: p3; whose RMM DQ6 rebounded more vigorously than did his initial iDSA non-RMM A3, p6; whose RMM B7 rebounded more vigorously than did his iDSA RMM A3 and p9; whose non-RMM B7 had a slightly higher MFI value than her initial iDSA RMM B35 (**Table S2, SDC**, http://links.lww.com/TXD/A689). Rebound in the first postoperative month was highest for RMM-DSA with the prior graft removed (transplantectomy n = 7, 86% of predesensitization MFI), lower for non-RMM-DSA (n = 30; 39% of predesensitization MFI) and lowest for RMM-DSA with the prior graft in situ (n = 5, *P* = 0.018 for RMM-DSA transplantectomy versus RMM-DSA graft in situ, **Table S2 and Figure S4, SDC**, http://links.lww.com/TXD/A689).

Of 4 patients with class I DSA alone, 2 did not experience a rejection (p1 and p11), 1 rebounded his class I DSA during AMR with subsequent slow decrease in DSA with good and stable renal function at 82-mo follow-up (p6) and 1 patient experienced ca-AMR and lost his graft at 81 mo (p4, Table 4). All patients with (combined) class II DSA experienced rejection with more rebound of their class II DSA than of class I, except for 1 patient (p13) with vigorous rebound of both class I and class II at the time of her AMR (**Table S2, SDC**, http://links.lww.com/TXD/A689). Eight of 11 patients with AMR proceeded to early ca-AMR at median 15 mo after transplantation (range 5–56, median 11 mo after 5 PE versus median 24 mo after 10 PE, Table 4).

One patient died of myocardial infarction at 43 mo. Death-censored graft survival was 73% after a median follow-up of almost 5 y (median 59 mo). Four patients lost their graft because of ongoing ca-AMR at a median of 71 mo (Table 4).

## DISCUSSION

This patient series describes anti-HLA antibody kinetics before, during, and after PE desensitization and clinical outcomes in 16 living-donor XM-positive patients. Their HLA incompatibility was reflected in a positive FC-XM in all patients accompanied by a positive CDC-XM in 50% as well as in high MFI levels of cumDSA and iDSA (median 31 399 and 19 089, respectively). PE was only modestly effective with regard to a decrease in MFI values of DSA. In the anti-HLA antibody analysis including non-DSA, HLA class II MFI response to desensitization was lower than class I in all groups. Noteworthy, within HLA class II, MFI response to desensitization was the lowest for HLA-DQ in both groups 2 and 3 (data not shown).

These modest, especially for class II, desensitization rates are in line with other desensitization reports.^19,27-30^ In these reports with either PE, IA, or DFPP, class I reduction rates varied between 61% and 86%, whereas class II DSAs were reduced between 33% and 74%. Pinelli et al^20^ do report equal titer reductions for class I and II anti-HLA antibodies with PE and IVIg but observed a similar reduced efficacy of PE beyond 4 sessions. A novel means of desensitization, imlifidase, was equally effective for class I and II with high reduction rates of 93% and 95%, respectively.^31^

Several observations can be made from this descriptive desensitization series. First, the reduction in MFI by PE was lower for class II anti-HLA antibodies, including DSA, than for class I. The additional reduction between the fifth and the 10th PE was very modest. Second, predesensitization iDSAs were also the iDSA as measured directly after desensitization, and, importantly, also the iDSA at time of ABMR diagnosis in the majority of patients (73%). Third, the predesensitization iDSA was not a RMM at the antigen level in the majority of patients (only in 29%). In 3 patients, a different HLA specificity became immunodominant at time of AMR, and in 2 of them, rebound of RMM was responsible for this change in immunodominance. Overall, RMM-DSA corresponding to grafts with transplantectomy rebounded more than RMM-DSA shared with the prior graft in situ. Fourth, of 8 patients with a positive CDC-XM before desensitization, 7 had MFI class II DSA of >17 000. Noteworthy, 5 of these were of DQ class, all with MFI of >20 000. With such high MFI values, the possibility of bead saturation hampering accurate antibody assessment should be considered according to Sensitization in Transplantation: Assessment of Risk recommendations. Results in undiluted samples should be approached cautiously, and therefore, we performed serial dilution of predesensitization sera.^20,32,33^

Although dilutions revealed higher titer class II antibodies than class I antibodies, there was no difference in titer between the DSA in the patients with failed desensitization because of a remaining positive CDC-XM, versus the DSA in patients who had converted their CDC-XM upon desensitization.

Another observation was that the majority of newly detected DSA detected within 1 mo were directed at repeated mismatches at the antigen or epitope level. It can be hypothesized that these newly detected DSAs can result from alloimmune memory responses, in line with the Sensitization in Transplantation: Assessment of Risk recommendations.^32^ All newly detected DSAs remained lower than the (predesensitization) iDSA.

Senev et al^34^ also underscored the importance of the class and the MFI value of the pretransplant iDSA, with the persisting DSA after kidney transplantation being more often of class II, especially of the DQ locus. In line with our findings, both Fidler et al^35^ and Bentall et al^36^ found class II DSA to be associated with inferior graft and patient outcomes. In immunized kidney transplant candidates opting for repeat transplantation, especially anti-HLA-DQ antibodies can be challenging.^37^ Indeed, all patients except 1 retransplant patient in our cohort had anti-HLA-DQ antibodies.

We were not able to include HLA-DP DSA in our analyses in these 16 cases mainly because of the lack of HLA-DP typing of the donors during this study time which precluded DSA determination. Nevertheless, predesensitization HLA-DP bead reactivity was detected in 11 (69%) of 16 cases. Given the fact that HLA-DP antibodies can have a deleterious effect on transplant outcomes,^38^ studies including HLA-DP antibody analyses are warranted.

CDC-XM was the decisive test to proceed with transplantation in our program, and DSA, albeit at a relatively lower MFI, remained present at the time of transplantation despite desensitization. AMR was very frequent (85%) accompanied by very high cumDSA and iDSA posttransplant and was only absent in 2 patients with class I DSA without rebound and a predesensitization-negative CDC-XM. Subsequent ca-AMR occurred frequently and was diagnosed early at median 15 mo.

Our AMR rate was much higher than in other reports on HLAi living-donor kidney transplantation, with AMR rates between 7% and 50%.^28,36,39-42^ Despite the high ABMR rate in our cohort, the death-censored graft survival of 73% after 5 y of median follow-up is in line with other reports, despite early ca-AMR.^36,39,43^ A possible explanation for the high AMR rate is that in our cohort the MFI value of the iDSA was very high and all had a positive FC-XM, whereas other studies either report lower predesensitization MFI values or also contained patients with a virtual XM only. We performed a CDC-XM without anti-human globulin-enhancement and with unseparated T and B cells, which may have resulted in underestimation of anti-HLA class II antibodies. Another consideration in the interpretation of the DSA measurement is that this is a cohort of highly sensitized patients with multiple bead reactivities, and therefore, we could not exclude that epitopes were shared between different beads. This could have led to underestimation of the DSA strength.^32,44,45^

Rituximab, or another B cell–depleting agent, was not part of our desensitization protocol. Jackson et al^46^ compared desensitization with and without rituximab and found less DSA rebound after rituximab induction, however, with no effect on DSA elimination, AMR and graft survival. The more intense rebound of RMM, at least in RMM-DSA corresponding to the previously removed allograft, than non-RMM in our cohort points to the relevance of HLA-specific memory B cells, as described by Wehmeier et al.^47^ The lower rebound of RMM-DSA corresponding to prior grafts remaining in situ is an interesting finding and needs validation in a larger cohort.

Although graft outcomes are inferior compared with transplantation in the absence of DSA, patient survival is favorable compared with remaining on dialysis.^8,48^ Also in our cohort, with enhanced immunosuppression because of frequent ABMR, patients survival was overall good. Desensitization therefore remains a viable option for a disadvantaged group of patients with very low chances of finding an HLA-compatible donor. In highly sensitized patients, transplant rates sharply decrease in patients approaching PRA of 100%. Schinstock et al^49^ demonstrated that in the United States, transplant rates in cPRA 99.5%–99.6% were 4 times higher than in cPRA ≥99.9%, the highest end PRA level that was present in 63% of our cohort. Different from our cohort, living-donor transplantation made up only 2.5% of transplantations in this 100% cPRA group.

The inferior removal of class II anti-HLA antibodies and their frequent immunodominance in DSA should be taken into account while considering allocation options for desensitization candidates. Donor selection, or delisting unacceptable antigens, is of utmost importance to avoid failed desensitization and rebound of DSA with consequent AMR and accelerated graft loss.^50^ The 3-y follow-up data after imlifidase desensitization are in line with the immunodominance data in our cohort: in a study by Kjellman et al,^43^ class II DSAs were more often immunodominant than class I, with higher class II pre-imlifidase levels and higher class II rebound. The high AMR and ca-AMR rate in our cohort stresses the need for the continuous search for a donor with a more favorable HLA type than the intended donor.

Our patient series has the overt limitation of a small sample size. Desensitization for CDC-XM combinations is a last resort option for patients highly unlikely to receive a compatible kidney transplant. Another limitation is heterogeneity in immunization, as 4 patients were combined ABO-incompatible and HLAi. Although relevant for comparing clinical outcomes, for the MFI decrease of DSA after PE, this was irrelevant, as induction was only administered after the last PE. DSAs were monitored directly before the last PE, and therefore, actual MFI values at transplant were lacking. Another limitation is that we were not able to analyze the HLA-DP DSA because of lack of HLA-DP typing of the donor in the majority of the cases. The strength of this study is the information on immunizing events and the median follow-up of 59 mo.

In conclusion, PE has a modest effect on anti-HLA antibody MFI reduction, especially of class II. In donor selection for desensitization candidates, class II immunodominance should be avoided as much as possible.

## ACKNOWLEDGMENTS

The authors thank the HLA typing and screening laboratory in Leiden, The Netherlands, for technical assistance.

## Supplementary Material


