# Supplementary material for: Anti-HLA Class II Antibodies Are the Most Resistant to Desensitization in Crossmatch-positive Living-donor Kidney Transplantations: A Patient Series
Source: Transplant Direct. 2024 Aug 29;10(9):e1695. doi: 10.1097/TXD.0000000000001695 (PMC11365629; doi:10.1097/TXD.0000000000001695)
Supplement: Supplementary file 1 [file txd-10-e1695-s001.pdf]

SUPPLEMENTAL DIGITAL CONTENT (SDC)

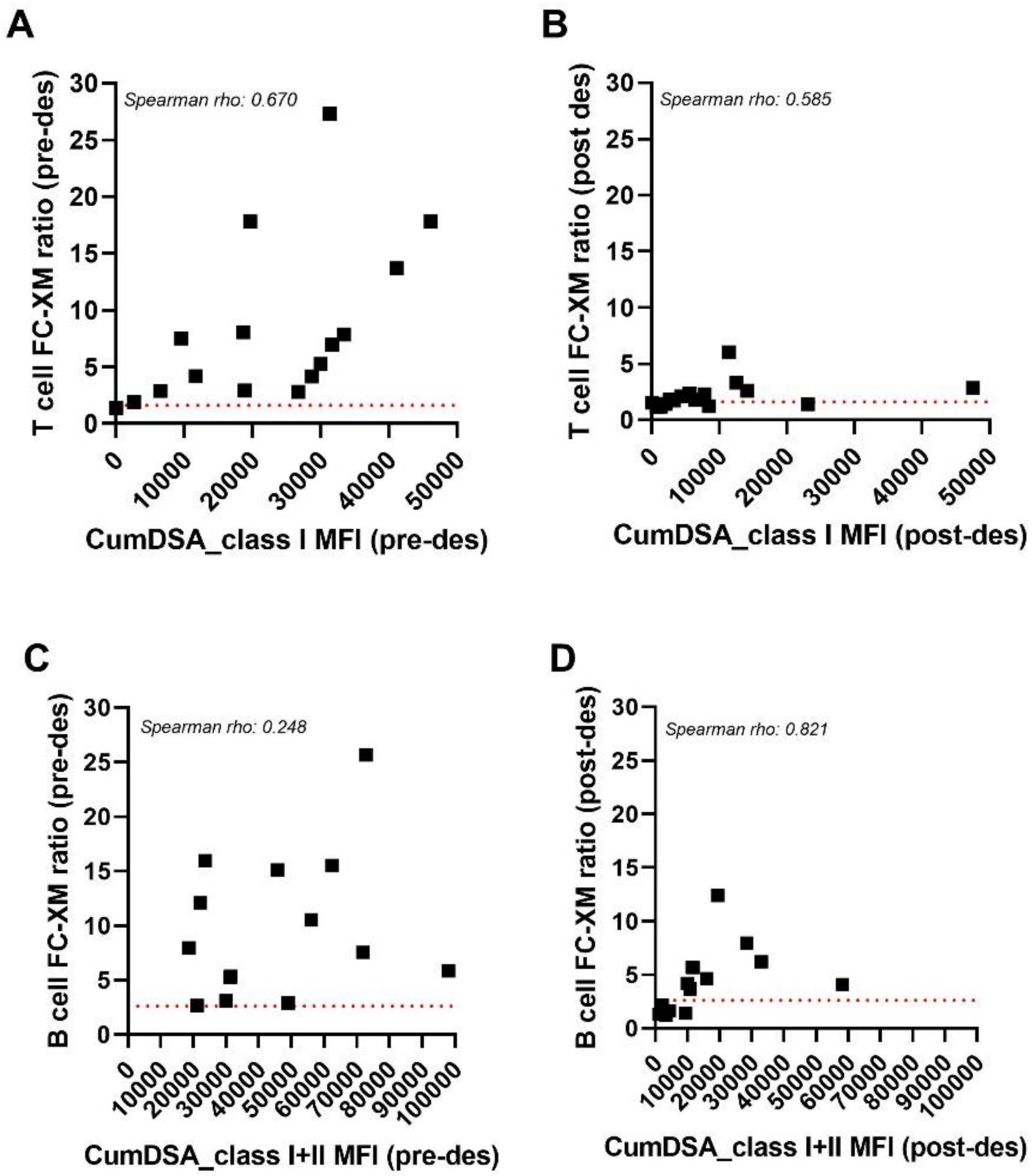

SDC, Figure S1. Correlation of cumDSA MFI with as compared to A-B) T and C-D) B cell FC-XM ratios before (pre-des) and after desensitization (post-des).

FC-XM: flow cytometric-crossmatch, CumDSA: cumulative DSA, MFI: Mean fluorescence intensity

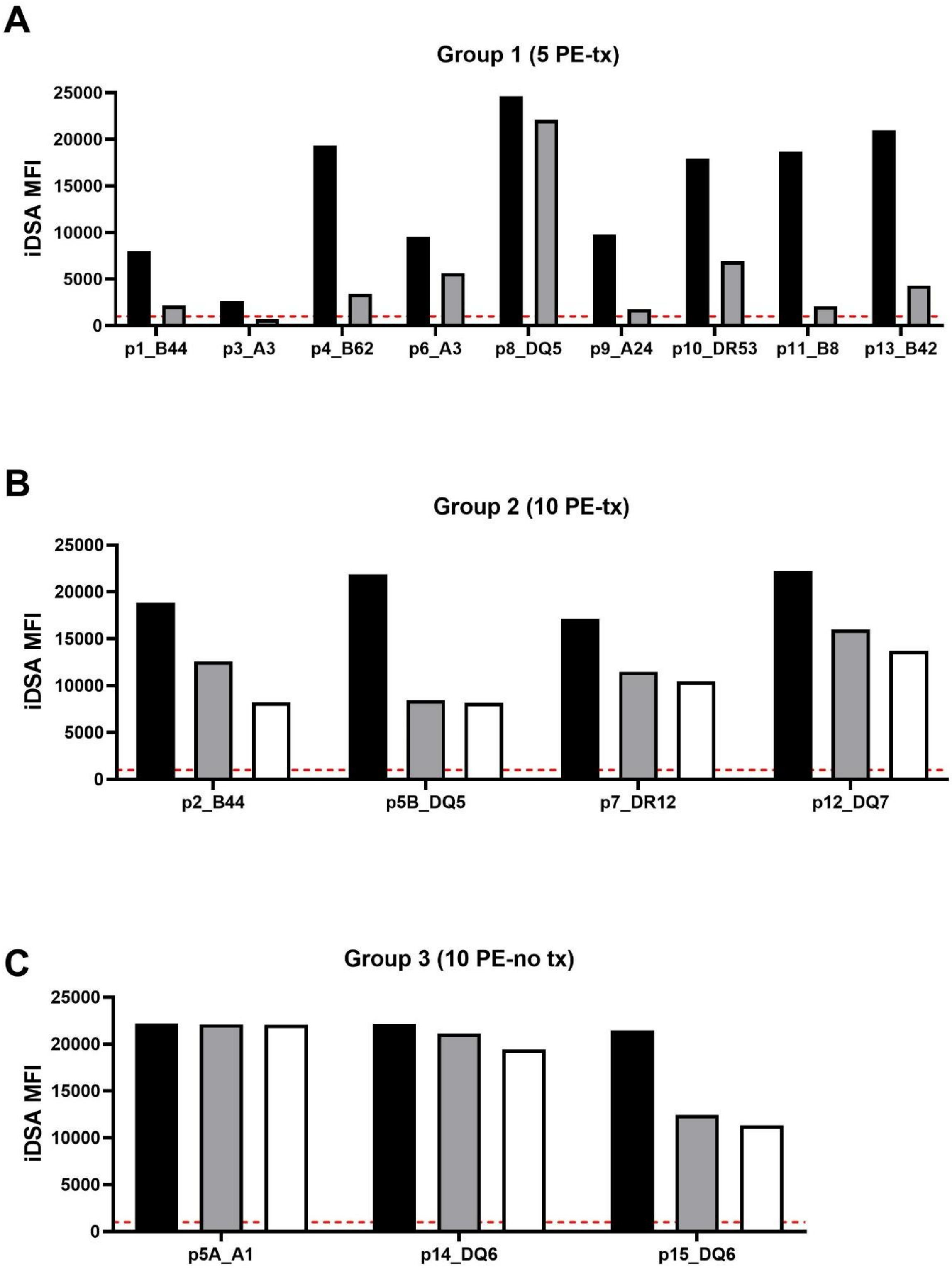

A
Group 1 (5 PE-tx)
iDSA MFI
25000
20000
15000
10000
5000
0
p1_B44
p3_A3
p4_B62
p6_A3
p8_DQ5
p9_A24
p10_DR53
p11_B8
p13_B42
B
Group 2 (10 PE-tx)
iDSA MFI
25000
20000
15000
10000
5000
0
p2_B44
p5B_DQ5
p7_DR12
p12_DQ7
C
Group 3 (10 PE-no tx)
iDSA MFI
25000
20000
15000
10000
5000
0
p5A_A1
p14_DQ6
p15_DQ6

SDC, Figure S2: Changes in the immunodominant DSA during desensitization: Patients were divided in three groups according to desensitization outcome: 5 PE and transplanted (group 1, n=9); 10 PE and transplanted (group 2, n=4) and 10 PE and not transplanted (group 3, n=3). For each patient, immunodominant DSAs are depicted. **A)** In group 1, iDSA was directed at HLA-A in 3 patients, -B in 4, HLA-DR in 1 and -DQ in 1 patient. Eight out of 9 patients had an iDSA MFI greater than 41% before the 5th PE, with the lowest decrease of 10% for DQ5 (p8). One patient (p10) converted from positive to negative CDC-XM before the 5th PE; others remained negative. **B)** In group 2, iDSA was directed at HLA-B in 1, HLA-DR in 1 and HLA-DQ in 2 patients. The median iDSA MFI decrease was 33% before the 5th PE and only minimal (11%) between the 5th and 10th PE. **C)** In group 3, iDSA was directed at HLA-A in 1 and HLA-DQ in 2 patients. The decrease in iDSA by PE was minimal with only 4% median decrease before the 5th PE and only 8% additional decrease between the 5th and 10th PE. All group 3 patients remained CDC-XM positive before the 10th PE. The black bars represent MFI before the 1st PE, the grey bars before the 5th PE and the white bars before the 10th PE.

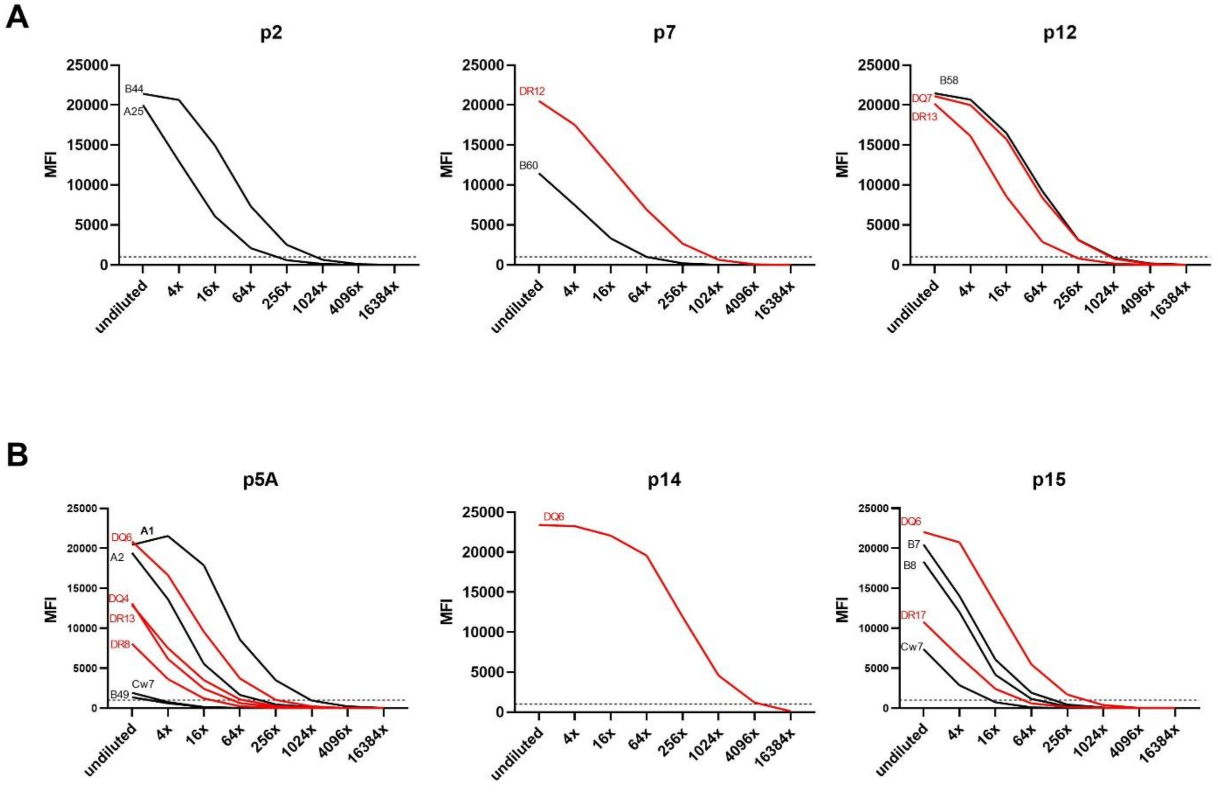

SDC, Figure S3: Kinetics of single DSA in each patient upon dilution in pre-desensitization sera. All patients in group 2 and group 3 were CDC-XM positive before desensitization. **A)** Group 2 (n=4) consisted of patients with 10 PE and a negative CDC-XM after desensitization and were transplanted. **B)** Group 3 (n=3) consisted of patients with 10 PE with a positive CDC-XM after desensitization and were not transplanted. Class I DSA are depicted in black, where class II DSA are depicted in red. High titer antibodies (>1024) in group 3 (n=4) consisted of 3 HLA-DQ6 DSA and 1 HLA-A1 DSA, while in group 2 (n=4) high titer DSA was directed at HLA-B 44 in one patient and HLA-B58, -DR12 and-DQ7 in the other one.

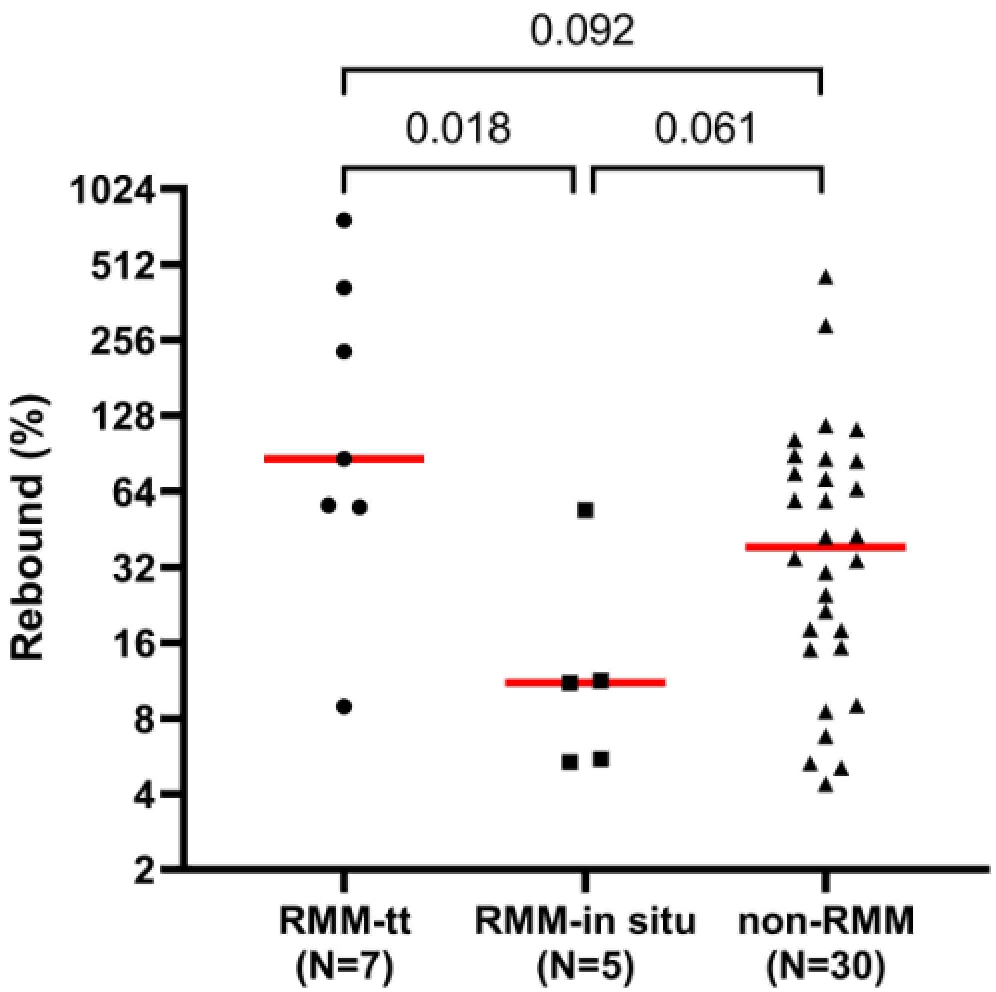

SDC, Figure S4: Rebound of repeated mismatches donor-specific antibodies. In the transplanted cohort (n=13), 43 donor-specific antibodies (DSA) were present; 13 DSA shared a repeated mismatch with a prior graft or child (RMM-DSA) and in 30 DSA no repeated mismatch could be established (no RMM-DSA).

DSA: donor-specific antibody; RMM-tt: DSA sharing a repeated mismatch with prior graft removed (transplantectomy); RMM-in situ: DSA sharing a repeated mismatch with graft in situ;
non-RMM: DSA that could not be linked to prior graft or pregnancy

SDC, Table S1: Characteristics of the four patients who underwent combined HLA- and ABO-incompatible kidney transplantation.

patient	XM and number of PE	donor	recipient	anti-A IgG titer			AMR
				pre-PE	pre-operative	maximum post-operative	
**p5**	CDC+ 2 wks PE	A	B	16	4	8	week 6 DSA rebound
**p7**	CDC+ 2 w PE	A	B	64	8	16	day 11 DSA rebound
**p9**	CDC- 5 PE	A	B	2	2	<2	week 3 DSA rebound
**p11**	CDC- 5 PE	A	O	32	4	<2	- (biopsy ATN, no C4d) No DSA

XM: crossmatch, PE: plasma exchange, CDC: complement-dependent cytotoxicity,
AMR: antibody-mediated rejection, DSA: donor-specific antibody, ATN: acute tubular necrosis

SDC, Table S2: MFI values for current and previous transplant donors/candidates and child mismatches. Repeated mismatches are in red script.

**Patient #1**	**pre-des**	**post-4th des**	**post-tx day 27**		**tx2- current donor**
**A33**	3723	1097	927		
**B44**	8007	2151	2785		
**Cw2**	874	196	451		
**DQ2**	0	0	123		
**Patient #1**	**pre-des**	**post-4th des**	**post-tx day 27**		**tx1 –donor *graft in situ***
**A31**	454	280	0		
**A32**	938	553	1021		
**B51**	191	39	204		
**B63**	589	406	921		
**Cw2**	874	196	451		
**DR13**	110	24	74		
**DQ6**	1664	463	1142		
**Patient #1**	**pre-des**	**post-4th des**	**post-tx day 27**		**child 1&2&3**
**A68**	7090	7775	111		
**B51**	191	39	204		
**Cw16**	0	30	75		
**DR4**	0	15	684		

**DR53**	1739	546	1969		
**DQ8**	650	225	688		

**Patient #2**	**pre-des**	**post-4th des**	**post-9th des**	**post-tx day 7**	**tx1- current donor**
**A25**	12484	5880	3198	3816	
**B44**	18847	12600	8205	7967	
**Cw5**	0	46	3	0	
**DR1**	0	54	32	0	
**DQ5**	0	0	0	0	

**Patient #3**	**pre-des**	**post-4th des**	**post-tx day 28**		**tx7- current donor**
**A3**	2657	671	12165		
**B7**	460	0	10040		
**DR13**	241	168	1343		
**DQ6**	2174	419	16705		
**Cw6**	1521	178	4568		**tx6-donor *transplantectomy***
**Cw15**	690	0	3625		
**B18**	0	0	1347		**tx5-donor *transplantectomy***
**Cw7**	113	0	593		

**A23**	1373	19	5086		**tx4-donor *transplantectomy***
**B49**	10038	5311	22702		
**B57**	12552	8471	21515		
**cw6**	1521	178	4568		
**B44**	7782	4115	15497		**tx3-donor *transplantectomy***
**Cw1**	12546	10607	16761		
**Cw5**	1879	521	2219		
**A11**	2749	683	18118		**tx2-donor *transplantectomy***
**DR12**	1037	433	11510		
**DR13**	241	168	1343		
**DQ6**	2174	419	16705		
**B27**	1252	165	15026		**tx1-donor *transplantectomy***
**B60**	1579	142	14686		
**Cw3**	9715	6313	13564		
**Cw1**	12546	10607	16761		
**DR13**	241	168	1343		

**Patient #4**	**pre-des**	**post-4th des**	**post-tx day 7**		**tx5- current donor**
**B18**	10693	982	9216		

**B62 (B15)**	19330	3417	16336		
**CW1**	247	27	145		
**DR1**	29	0	25		
**DR9**	862	42	1773		
**DQ5**	386	0	1413		
**DQ9**	20	0	147		
**B56**	20455	5568	17828		**tx4-donor** ***transplantectomy***
**CW4**	438	160	128		
**DR9**	862	42	1773		
**DQ9**	20	0	147		
**B27**	387	35	1047		**tx3-donor** ***graft in situ***
**CW2**	374	101	178		
**DR16**	11	0	107		
**DQ5**	386	0	1413		
**B44**	5311	287	9699		**tx2-donor** ***transplantectomy***
**DR8**	3951	702	15729		
**DQ4**	2448	547	112		
**A3**	21046	6028	21779		**tx1-donor** ***transplantectomy***
**B35**	19896	6845	21233		

**Cw4**	438	159	128		
**DR17**	4589	1376	17563		
**DR52**	2601	113	5613		
**DQ2**	17968	6252	16859		

**Patient #5B**	**pre-des**	**post-4th des**	**post-9th des**	**post-tx day 32**	**tx1- current donor**
**A24**	8266	2497	1978	1498	
**B18**	0	0	66	0	
**Cw7**	1308	723	681	1475	
**DR16**	90	195	807	114	
**DR51**	99	126	509	59	
**DQ5**	21893	8447	8174	15616	
**A26**	13953	7909	5254	17193	**Child**
**Cw12**	181	32	219	41	
**DR13**	10784	3679	2851	5728	
**DQ6**	22207	8949	9106	16741	

**Patient #6**	**pre-des**	**post-4th des**	**post-tx day 8**		**tx4- current donor**
**A3**	9543	5654	21929		
**B7**	5540	1917	22880		
**B44**	3826	900	11180		
**Cw5**	27	270	1825		
**DR12**	73	54	113		

**DR15**	72	176	1200		
**DR51**	2282	968	2660		
					**tx3-donor**<br>**no mismatches**<br>***transplantectomy***
**A2**	21451	21811	16749		**tx2-donor**<br>***transplantectomy***
**A68**	20002	15474	18770		
**B13**	4882	999	11746		
**B51**	10467	3320	19909		
**Cw6**	95	183	1737		
**Cw15**	1821	255	255		
**DR7**	7575	3108	20503		
**DR53**	20512	15150	20220		
**DQ2**	12961	6070	16788		
**A3**	9543	5654	21929		**tx1-donor**<br>***transplantectomy***
**B7**	5540	1917	22880		
**DR14**	333	494	629		

**Patient #7**	**pre-des**	**post-4th des**	**post-9th des**	**post-tx day 12**		**tx3-<br>current donor**
**A24**	124	113	199	1379		
**B60**	6529	2067	1305	3540		
**Cw10**	108	82	143	1327		

**DR12**	17173	11492	10478	11268		
**DR52**	905	624	690	2241		
**B60**	6529	2067	1305	3540		**tx2-donor** ***graft in situ***
**Cw10**	108	82	143	1327		
**DR13**	20518	19927	21130	22123		
**DR52**	905	624	690	2241		
**DQ6**	20884	21744	22134	22345		
**B13**	21822	20748	19675	22462		**tx1-donor** ***transplantectomy***
**B62**	12284	4223	2796	7224		
**Cw6**	19018	13474	11036	15493		
**Cw10**	108	82	143	1327		
**DQ8**	15463	11546	8261	13422		

**Patient #8**	**pre-des**	**post-4th des**	**post-tx day 14**		**tx4- current donor**
**A1**	13704	11276	7748		
**B57**	271	118	201		
**Cw6**	15005	11831	1022		
**DR1**	2396	1264	2455		
**DQ5**	24612	22069	22154		
**DQ9**	16177	11706	9009		
**A26**	20387	16620	6745		**tx3-donor** ***transplantectomy***

**Cw15**	20037	16755	2497		
**Cw16**	380	166	314		
**DR13**	3129	1833	2924		
**DQ6**	24538	21023	20827		
**A1**	13704	11276	7748		**tx2-donor *transplantectomy***
**B39**	1196	776	266		
**Cw5**	23410	19159	4922		
**DQ9**	16177	11706	9009		
**A3**	6233	4621	1159		**tx1-donor *transplantectomy***
**B44**	6964	5413	2678		
**Cw5**	23410	19159	4922		
**DR2**	2486	1245	2346		
**DQ1**	24728	21370	21307		

**Patient #9**	**pre-des**	**post-4th des**	**post-tx day 24**		**tx3- current donor**
**A3**	6048	956	929		
**A24**	9784	1793	499		
**B7**	8408	1951	1509		
**B35**	9172	1778	505		
**Cw4**	593	245	30		
**Cw7**	157	146	0		
**DR13**	5720	1722	251		

**DR52**	4342	1118	654		
**DQ6**	2301	623	984		
**B8**	13113	3318	5012		**tx2-donor *transplantectomy***
**DR3**	5610	1554	429		
**A2**	21164	9951	19653		**tx1-donor *graft in situ***
**A28**	20536	9127	19250		
**B35**	9172	1778	505		
**B60**	9225	2081	1974		
**Cw4**	593	245	30		
**DR1**	70	90	108		
**DQ5**	8686	2533	3863		

**Patient #10**	**pre-des**	**post-4th des**	**post-tx day 12**		**tx3- current donor**
**A68**	15259	4128	1843		
**B44**	12539	2882	2687		
**B35**	0	38	0		
**Cw4**	3863	796	205		
**DR7**	655	402	656		
**DR13**	2914	596	156		
**DR53**	17952	6921	1990		
**DQ6**	3610	675	326		
**A28**	15134	4041	1715		

**A31**	21289	11712	9028		**tx2-donor** ***graft in situ***
**B27**	4881	717	978		
**DR4**	2031	658	1029		
**DR13**	2914	596	156		
**DR53**	17952	6921	1990		
**A1**	21862	13495	13955		**tx1-donor** ***transplantectomy***
**B8**	14811	3257	2921		
**Cw7**	8155	2730	1179		

**Patient #11**	**pre-des**	**post-4th des**	**post-tx day 22**		**tx2- current donor**
**A3**	0	47	0		
**B8**	18677	2102	1590		
**DR15**	0	32	0		
**DQ6**	0	60	0		
**B7**	798	0	570		
**B57**	0	0	75		**tx1-donor** ***graft in situ***
**Cw6**	522	31	326		
**DR13**	0	177	0		
**DR15**	0	33	0		
**DR52**	0	201	0		

Patient #12	pre-des	post-4th des	post-9th des	post-tx day 27		tx3- current donor
**B58**	19687	16474	12526	6708		
**DR13**	20474	9816	6773	1827		
**DQ7 (3)**	22270	15988	13728	16767		
**B49**	23713	15782	12535	17070		**tx2-donor** ***transplantectomy***
**DR13**	20474	9816	6773	1827		
**DR15**	19007	7320	4345	10639		
**DQ6**	22493	16405	15688	19125		
**B38**	22556	19385	15593	17823		**tx1-donor** ***graft in situ***
**Cw12**	11451	4057	2170	7173		
**DR4**	19889	8326	5857	12248		

Patient #13	pre-des	post-4th des	post-tx day 14		tx1- current donor
**A3**	57	12	1052		
**B42**	20974	4296	18645		
**Cw17**	5756	1299	3389		
**DR11**	11140	2836	6576		
**DR52**	11172	2316	17175		
**B51**	27713	23011	23484		**child**
**Cw16**	5933	713	2979		
**DR17 (3)**	16167	5083	18484		

**DR52**	11172	2316	17175		
**DQ2**	7952	1372	5252		

**Patient #5A- no tx**	**pre-des**	**post-4th des**	**post-9th des**		**current donor candidate**
**A1**	22196	22115	22068		
**A2**	20429	11823	21412		
**B49**	1180	318	987		
**cw14**	516	456	381		
**cw7**	2355	826	3047		
**DR8**	6324	2766	4407		
**DR13**	11617	5454	8857		
**DQ4**	13391	5479	16649		
**DQ6**	20607	13879	21083		
**A26**	19659	12713	20314		
**Cw12**	631	579	438		**Child**
**DR13**	11617	5454	8857		
**DQ6**	20607	13879	21083		

**Patient #14- no tx**	**pre-des**	**post-4th des**	**post-9th des**		**current donor candidate**
**A2**	0	0	0		
**A25**	0	0	0		
**B18**	0	0	0		

**B62**	0	0	0		
**Cw10**	0	0	0		
**Cw12**	0	0	0		
**DR13**	999	337	182		
**DR15**	377	247	152		
**DR51**	354	306	247		
**DQ6**	22159	21163	19405		
**A3**	0	0	0		**tx2-donor**<br>***graft in situ***
**Cw10**	0	0	0		
**DR7**	2002	749	417		
**DR53**	1284	463	210		
**DQ9**	21587	21959	20375		
					**tx1-donor**<br>**no mismatches**<br>***transplantectomy***

**Patient #15- no tx**	**pre-des**	**post-4th des**	**post-9th des**		**current donor candidate**
**A1**	22	79	115		
**A26**	41	93	0		
**B7**	19742	7781	7327		
**B8**	17362	6563	5948		
**cw7**	4037	972	834		
**DR15**	178	193	130		
**DR17**	10245	3701	2981		
**DR51**	61	321	209		
**DQ2**	582	262	159		
**DQ6**	21464	12441	11320		
**A2**	4	38	27		**tx1 –donor** ***graft in situ***
**B60**	20327	10054	9419		
**Cw3**	9026	2294	2092		
**DR13**	7344	2286	1761		
**DQ6**	21464	12441	11320		
